# *APOE*‐guided lecanemab dosing dissociates ARIA‐E from ARIA‐H: The K‐ROAD study

**DOI:** 10.1002/alz.71765

**Published:** 2026-08-25

**Authors:** Heekyoung Kang, Hyemin Jang, Jehyun Ahn, Duk L Na, Jae‐Hong Lee, Eun‐Joo Kim, Min Young Chun, Soo Hyun Cho, Byeong C. Kim, Si Eun Kim, Gyeongmo Sohn, Jihwan Yun, Jin San Lee, Key‐Chung Park, Hee Kyung Park, Young Ju Kim, Kyungbok Lee, Sohyun Yim, Seonghyeon Kim, Jun Pyo Kim, Hee Jin Kim, Jae‐Sung Lim, Sang Won Seo

**Affiliations:** ^1^ Department of Neurology, Samsung Medical Center Sungkyunkwan University School of Medicine Gangnam‐gu Seoul Republic of Korea; ^2^ Department of Neurology Soonchunhyang University Seoul Hospital, Soonchunhyang University School of Medicine Seoul Republic of Korea; ^3^ Department of Neurology, Asan Medical Center University of Ulsan College of Medicine Seoul Republic of Korea; ^4^ Happymind Clinic Seoul Republic of Korea; ^5^ Department of Neurology Pusan National University Hospital Busan Republic of Korea; ^6^ Department of Neurology Yonsei University College of Medicine Seoul Republic of Korea; ^7^ Department of Neurology Yongin Severance Hospital, Yonsei University Health System Yongin‐si Gyeonggi‐do Republic of Korea; ^8^ Department of Neurology Chonnam National University Hospital, Chonnam National University Medical School Gwangju Republic of Korea; ^9^ Department of Neurology Inje University Haeundae Paik Hospital Busan Republic of Korea; ^10^ Department of Neurology Kyung Hee University Medical Center, Kyung Hee University College of Medicine Seoul Republic of Korea; ^11^ Alzheimer's Disease Convergence Research Center Seoul Republic of Korea; ^12^ Department of Neurology Hallym University Chuncheon Sacred Heart Hospital Chuncheon Republic of Korea; ^13^ Neuroscience Center Seoul Republic of Korea; ^14^ Department of Digital Health SAIHST, Sungkyunkwan University Seoul Republic of Korea

**Keywords:** Alzheimer's disease, amyloid‐related imaging abnormalities, apolipoprotein E, cerebral microbleeds, dose escalation, genotype‐guided dosing, lecanemab

## Abstract

**INTRODUCTION:**

We evaluated whether apolipoprotein E (*APOE* εε4) genotype‐guided lecanemab escalation mitigates the *APOE* εε4–driven amyloid‐related imaging abnormalities (ARIA) with edema (ARIA‐E) gradient and whether ARIA‐E and ARIA with hemosiderin deposition (ARIA‐H) are mechanistically distinct.

**METHODS:**

Five hundred eight patients with early Alzheimer's disease received *APOE* genotype‐guided lecanemab escalation (target maximum plasma concentration [Cmax] thresholds: 118, 308, and 404 µg/mL for εε4/εε4, heterozygote, and non‐carrier) across eight Korean centers, with magnetic resonance imaging‐based ARIA surveillance.

**RESULTS:**

ARIA‐E incidence was 2.0% in non‐carriers and 6.2% in εε4/εε4 homozygotes (εε4/εε4‐versus‐non‐carrier OR 3.25, versus 8.49 in Clarity AD and 8.32 in Japan post‐marketing surveillance). Any ARIA‐H was independently predicted by baseline cerebral microbleeds, white matter hyperintensity (WMH), age, and *APOE* εε4 allele dose (aORs 1.23, 1.50, 1.04, 1.75).

**DISCUSSION:**

Under genotype‐guided escalation, observations are compatible with – but do not establish – attenuation of the *APOE* εε4‐driven ARIA‐E gradient, whereas ARIA‐H risk reflected baseline cerebrovascular burden. These findings raise the hypothesis of a dual‐axis safety framework requiring randomized confirmation.

## Highlights

- Observations are compatible with attenuation of the *APOE* εε4‐driven ARIA‐E gradient under titration.
- Any ARIA‐H reflected baseline cerebrovascular burden in within‐cohort multivariable models.
- Lobar but not deep microbleeds predicted ARIA‐H, implicating CAA.
- Findings frame a dual‐axis safety framework as a working hypothesis.

## 1 BACKGROUND

Lecanemab is a humanized IgG1 monoclonal antibody targeting soluble amyloid beta (Aβ) protofibrils. In the Clarity AD trial, lecanemab demonstrated amyloid clearance and modest slowing of clinical decline, leading to regulatory approvals for early Alzheimer's disease (AD).^1^ Amyloid‐related imaging abnormalities (ARIA) represent a class effect of anti‐amyloid immunotherapies that is mechanistically inseparable from amyloid clearance.^1^, ^2^, ^3^, ^4^ ARIA includes ARIA with edema (ARIA‐E) and ARIA with hemosiderin deposition (ARIA‐H). The principal clinical challenge is therefore not avoidance but risk stratification through monitoring and titration protocols matched to each patient's vulnerability.

The disproportionate ARIA‐E risk in apolipoprotein E (*APOE* εε4) homozygotes has been a critical barrier to safe treatment. Recent pharmacokinetic analyses identified maximum plasma concentration (Cmax) as the primary exposure metric driving ARIA‐E risk, with genotype‐specific thresholds lowest in εε4/εε4 homozygotes.^5^, ^6^ Under standard flat dosing at 10 mg/kg biweekly, εε4/εε4 homozygotes exceed their threshold from the first infusion. This leaves no window for cerebrovascular adaptation. These findings provide a rationale for genotype‐guided dose escalation. Treatment is initiated at lower doses and gradually titrated upward, reducing peak exposure while preserving cumulative exposure and efficacy. Proof of concept was demonstrated in the TRAILBLAZER‐ALZ 6 trial. A modified donanemab titration schedule reduced ARIA‐E incidence at 24 weeks from 24.0% to 14.0% overall, with the largest reduction in *APOE* ε4 homozygotes.^7^

In contrast, the determinants of ARIA‐H remain less well characterized. ARIA‐H comprises new cerebral microbleeds (CMBs), cortical superficial siderosis (cSS), or macrohemorrhage.^3^ It may occur concurrently with ARIA‐E or as isolated ARIA‐H in the absence of concurrent ARIA‐E. Across anti‐amyloid trials, ARIA‐H rates have shown a less consistent relationship with drug exposure than ARIA‐E.^3^, ^8^ They instead correlate strongly with baseline cerebrovascular burden, including CMB count, white matter hyperintensities (WMHs), age, and underlying cerebral amyloid angiopathy (CAA). Isolated ARIA‐H, which by definition is unconfounded by concurrent ARIA‐E, may particularly directly reflect this pre‐existing vascular vulnerability.^9^, ^10^, ^11^ Mechanistically, ARIA‐H is hypothesized to reflect drug‐induced perturbation of a chronic structural vascular substrate.^12^, ^13^, ^14^ This contrasts with the acute, Cmax‐dependent vascular permeability changes that characterize ARIA‐E.^3^ Although *APOE* ε4 allele dose contributes to ARIA‐H risk, this association is weaker than for ARIA‐E and less clearly attenuated by reduced drug exposure.^1^ Therefore, whether titration‐based mitigation applies equally to ARIA‐H, including its isolated form, remains unresolved.

Here, we report the safety outcomes from the Korea‐Registries to Overcome Dementia and Accelerate Dementia Research (K‐ROAD) disease‐modifying therapy (DMT) subcohort. K‐ROAD is the first real‐world implementation of genotype‐guided lecanemab dose escalation. We evaluated two questions framed by a single intervention. First, does *APOE* genotype‐guided escalation mitigate the *APOE* ε4–driven ARIA‐E risk observed in Clarity AD? Second, does ARIA‐H (including its isolated form) exhibit a parallel response or an altogether distinct, titration‐independent risk pattern? Together, these questions test whether ARIA‐E and ARIA‐H require a unified or dual‐axis safety framework.

**RESEARCH IN CONTEXT**

1. **Systematic review**: The authors reviewed PubMed and conference abstracts on lecanemab safety. Whether *APOE* genotype‐guided escalation can mitigate the disproportionate ARIA‐E risk in εε4 homozygotes in real‐world practice and whether ARIA‐E and ARIA‐H reflect distinct mechanisms remain unresolved.
2. **Interpretation**: Our findings are compatible with – but do not statistically establish – attenuation of the *APOE* εε4‐dependent ARIA‐E gradient under genotype‐guided escalation. ARIA‐H risk reflected baseline cerebrovascular vulnerability – predominantly CAA‐related – independent of titration in within‐cohort analyses. These observations are hypothesis‐generating and frame a dual‐axis safety framework integrating *APOE*‐guided titration with MRI‐based screening for CAA‐related vascular burden (lobar microbleeds and moderate to severe WMHs).
3. **Future directions**: Beyond the converging cross‐cohort and within‐study evidence presented here, a randomized titration trial – analogous to TRAILBLAZER‐ALZ 6, in which titration intensity is varied independently of *APOE* genotype – would provide the definitive test of titration‐mediated modification of ARIA risk.

## 2 METHODS

### 2.1 Study design and setting

This was a multicenter observational cohort study conducted within the K‐ROAD DMT subcohort. The cohort was designed to bridge the evidence gap between randomized trials and post‐marketing surveillance (PMS) by combining real‐world dosing practices with clinical trial‐level monitoring rigor. Enrollment was prospective at the coordinating center (Samsung Medical Center) under a dedicated Institutional Review Board (IRB)‐approved protocol. Participating centers obtained IRB approval for retrospective ascertainment of clinical and imaging data collected under the same harmonized data‐collection and MRI surveillance standards. Seven university‐affiliated hospitals and one specialized memory clinic in Korea participated: Samsung Medical Center, Asan Medical Center, Happy Mind Clinic, Pusan National University Hospital, Yongin Severance Hospital, Chonnam National University Hospital, Haeundae Paik Hospital, and Kyung Hee University Hospital. The study protocol was approved by the IRB at each participating center (Samsung Medical Center, IRB No. 2024‐12‐050, 2025‐12‐011; Asan Medical Center, IRB No. 2025‐1550; Happy Mind Clinic, IRB No. P01‐202602‐01‐041; Pusan National University Hospital, IRB No. 2601‐034‐159; Yongin Severance Hospital, IRB No. 9‐2025‐0261; Chonnam National University Hospital, IRB No. CNUH‐2026‐027; Haeundae Paik Hospital, IRB No. 2026‐01‐005; Kyung Hee University Hospital, IRB No. 2026‐01‐068, and all participants or their legal representatives provided written informed consent. The study followed the STrengthening the Reporting of OBservational studies in Epidemiology (STROBE) reporting guideline.^15^

### 2.2 Participants

Participants were adults diagnosed with mild cognitive impairment (MCI) due to AD or mild AD dementia who were amyloid‐positive on positron emission tomography (PET)^16^ and initiated lecanemab between October 2024 and December 2025. The analysis cohort comprised 508 amyloid‐positive patients who initiated lecanemab and had follow‐up data through either the seventh biweekly infusion or earlier treatment discontinuation, whichever occurred first – comprising 487 patients (95.9%) who completed ≥7 infusions and 21 (4.1%) who discontinued earlier (10 owing to ARIA, seven to infusion‐related reactions [IRRs], and four for other clinical reasons). Eligibility followed the Korea Ministry of Food and Drug Safety prescribing information and the Korean Dementia Association Appropriate Use Recommendations,^17^ which classify findings such as more than four CMBs (17/505 [3.4%]), cSS (2/503 [0.4%]), and anticoagulant medication use (16/508 [3.1%]) as cautionary considerations rather than absolute exclusion criteria, thereby broadening cohort representativeness relative to Clarity AD. A small proportion of cognitively unimpaired amyloid‐positive individuals (*n* = 22; 4.3%) were also enrolled.

### 2.3 Genotype‐guided dose escalation

All participants underwent *APOE* genotyping prior to treatment initiation. A reference escalation protocol, developed at Samsung Medical Center, recommended individualized initial dosing based on *APOE* ε4 status: ε4/ε4 homozygotes started at 2.5 mg/kg with stepwise escalation to 10.0 mg/kg by the fourth infusion; ε4 heterozygotes with baseline CMBs started at 5.0 mg/kg; and ε4 heterozygotes without CMBs and non‐carriers received standard 10.0 mg/kg biweekly. As a real‐world multicenter cohort, participating centers adapted this protocol to local clinical practice based on institutional experience, treating physician judgment, and patient‐specific cerebrovascular burden. Actual mean administered mg/kg doses across infusions V1–V7 for each of the eight participating centers are presented in eTable S1, documenting the real‐world implementation of the protocol. All 64 ε4/ε4 homozygotes (100%) received reduced initial dosing (≤5 mg/kg at V1, below the standard 10 mg/kg), although actual subsequent escalation trajectories varied across centers within the constraints of the genotype‐stratified protocol. Both center‐level and within‐genotype sources of variation were subsequently addressed in the statistical analyses (Methods 2.8). Pharmacokinetic (PK) analyses used actual administered doses to reflect individual drug exposure.

### 2.4 Infusion‐related reaction monitoring

The IRRs were defined as adverse events occurring during or within 24 h after a lecanemab infusion and were graded using the Common Terminology Criteria for Adverse Events (CTCAE), version 5.0.^18^ Pre‐infusion premedication (most commonly acetaminophen, with antihistamine or low‐dose corticosteroid added at clinician discretion) was concentrated at the first two infusions and stepped down with treatment tolerance. Vital signs and clinical symptoms were monitored throughout each infusion and during a structured post‐infusion observation period.

### 2.5 Magnetic resonance imaging protocol and ARIA monitoring

Brain magnetic resonance imaging (MRI) including susceptibility‐weighted imaging (SWI) sequences was performed at all participating centers (eTable S2). Scheduled MRI assessments were performed before the fifth infusion (visit 5 [V5]) and before the seventh infusion (V7) at all eight centers. Upon detection of ARIA at any scheduled scan, follow‐up MRI was uniformly performed at all centers per the AD and Related Disorders Therapeutics Working Group (ADRD‐TWG) Appropriate Use Recommendations^19^ and Korean Dementia Association guidelines^17^ to confirm ARIA‐E resolution and ARIA‐H stability before resuming lecanemab dosing. ARIA‐E was defined as new or increased signal abnormality on fluid‐attenuated inversion recovery (FLAIR) imaging consistent with vasogenic edema, sulcal effusion, or both. ARIA‐H was defined as new CMBs, new or worsened cSS, or macrohemorrhage on SWI.^3^, ^8^ Isolated ARIA‐H was defined as ARIA‐H occurring without concurrent or preceding ARIA‐E. Radiologic and clinical severity of ARIA events were graded according to the ADRD‐TWG Appropriate Use Recommendations.^19^ All ARIA events were adjudicated by agreement between the treating neurologist and a neuroradiologist at each center.

Baseline WMH burden was assessed using the Fazekas scale, with deep WMH (D) and periventricular WMH (P) each scored from 0 to 3, and the summed score (D + P; range: 0 to 6) used as a continuous measure of WMH burden.^20^ CMBs were further classified by anatomical location as deep (basal ganglia, thalamus, brainstem) or lobar (cortical‐subcortical, including external capsule) per Microbleed Anatomical Rating Scale (MARS) criteria.^21^

### 2.6 Reference cohorts for cross‐study comparison

Incidence rates and odds ratios (ORs) for IRR, ARIA‐E, and ARIA‐H in K‐ROAD were compared with published data from the Clarity AD trial and the Japan PMS program.^1^, ^21^ MRI sequences differed across cohorts: K‐ROAD used SWI, whereas Clarity AD used gradient‐recalled echo (GRE)‐predominant protocols.^22^, ^23^

### 2.7 Pharmacokinetic simulation

Individual Cmax values were estimated using a published two‐compartment population PK model.^5^, ^6^ The central volume of distribution (V_1_) was calculated as V_1_ = 3.24 × (body weight/72)^0.513^, and Cmax was approximated as dose/V_1_. The genotype‐specific target Cmax thresholds used to inform the *APOE*‐stratified titration design (118, 308, and 404 µg/mL for *APOE* ε4/ε4 homozygotes, ε4 heterozygotes, and non‐carriers, respectively) correspond to the steady‐state Cmax associated with a 10% predicted ARIA‐E probability per *APOE* ε4 genotype, as derived from the published population pharmacokinetic/exposure‐response analysis of the lecanemab clinical development program.^5^ Albumin levels were not available but were shown in prior analyses to have minimal impact on lecanemab pharmacokinetics. Anti‐drug antibody (ADA) status was not measured in this real‐world cohort, and reliable individual‐level information regarding its potential effect on lecanemab exposure was therefore unavailable.

### 2.8 Statistical analysis

Baseline characteristics were summarized as means (standard deviations [SDs]) or medians (interquartile ranges [IQRs]) for continuous variables and as frequencies (percentages) for categorical variables. Group differences were tested with the Mann‐Whitney U test or Kruskal‐Wallis test for continuous variables and the χ^2^ or Fisher exact test for categorical variables, as appropriate. Incidence rates for IRR, ARIA‐E, and ARIA‐H were calculated for the overall cohort and stratified by *APOE* ε4 genotype. The ORs for ARIA‐E and for any ARIA‐H in ε4/ε4 homozygotes versus non‐carriers were computed within K‐ROAD and, using published counts, for reference cohorts (Japan PMS and Clarity AD). Given the modest size of the ε4/ε4 stratum (*n* = 64, with four ARIA‐E events expected at non‐carrier‐equivalent baseline rate), a post hoc detectable‐effect analysis was conducted to contextualize null findings.

Multivariable logistic regression used any ARIA‐H as the primary outcome and isolated ARIA‐H as a secondary outcome. Candidate covariates were evaluated by univariate logistic regression with any ARIA‐H as the reference outcome; variables with *p* < 0.1 were carried forward into the multivariable model. The same covariate set was applied to isolated ARIA‐H as parallel comparisons. Each model included baseline CMB count (per microbleed), WMH (summed Fazekas score^20^ [deep WMH 0 to 3 plus periventricular WMH 0‐3; range, 0 to 6], modeled as a continuous variable with effects expressed per 1‐point increase),^24^, ^25^ age (per year), and *APOE* ε4 allele dose (0, 1, or 2 ε4 alleles; additive genetic model). Effects were reported as adjusted ORs (aORs) with 95% CIs.

To account for potential site‐level confounding arising from heterogeneous titration practices across centers, multivariable models for ARIA‐H were re‐fit using cluster‐robust standard errors with clinical site (eight centers) as the clustering variable, and site‐stratified ARIA‐E and ARIA‐H incidences were additionally compared across centers using Mantel‐Haenszel estimators, with inter‐center heterogeneity quantified by the Breslow‐Day test. In a separate sensitivity analysis, lobar CMB count and deep CMB count were each substituted in turn for total CMB count in the multivariable model for ARIA‐H, to examine the location‐specific contribution of putative CAA‐related (lobar) versus hypertensive arteriopathy‐related (deep) microbleeds.

Analyses were performed using Python (version 3.10) with statsmodels (version 0.14) and SciPy (version 1.10). Two‐sided *p* values less than 0.05 were considered statistically significant.

## 3 RESULTS

### 3.1 Study population

A total of 508 patients across eight centers were included (Table 1). The mean (SD) age was 71.1 (8.5) years, 334 (65.7%) were female, and the mean (SD) body weight was 58.5 (10.1) kg. By diagnosis, 297 (58.5%) had MCI, 189 (37.2%) had mild AD dementia,^26^, ^27^ and 22 (4.3%) were cognitively unimpaired. *APOE* ε4 genotype distribution was 199 non‐carriers (39.2%), 245 heterozygotes (48.2%), and 64 homozygotes (12.6%). Baseline CMBs were present in 156 (30.9%), and the median Fazekas D+P sum was 2 (IQR, 2 to 3).

**TABLE 1:** Baseline characteristics of K‐ROAD DMT subcohort (*N* = 508).

Characteristic	Overall (*N* = 508)	Non‐carrier (*n* = 199)	Heterozygote (*n* = 245)	Homozygote (*n* = 64)
Age, mean (SD), years	71.1 (8.5)	70.7 (10.3)	72.0 (6.9)	68.5 (6.8)
Female sex, No. (%)	334 (65.7)	120 (60.3)	168 (68.6)	46 (71.9)
Education, mean (SD), years	13.4 (3.9)	13.5 (3.6)	13.2 (4.1)	13.4 (4.1)
Body weight, mean (SD), kg	58.5 (10.1)	59.7 (10.3)	57.9 (10.0)	57.2 (9.9)
MMSE, mean (SD)	23.4 (4.2)	23.0 (4.6)	23.7 (3.9)	23.3 (4.5)
CDR‐SB, mean (SD)	3.2 (2.1)	3.5 (2.3)	2.9 (2.0)	3.0 (1.9)
Clinical Dx, CU/MCI/mild AD, No.	22 / 297 / 189	8 / 109 / 82	11 / 147 / 87	3 / 41 / 20
Vascular risk factors				
Hypertension, No. (%)	194 (38.2)	78 (39.2)	100 (40.8)	16 (25.0)
Diabetes mellitus, No. (%)	101 (19.9)	49 (24.6)	44 (18.0)	8 (12.5)
Dyslipidemia, No. (%)	297 (58.5)	111 (55.8)	151 (61.6)	35 (54.7)
Medications				
Antiplatelet only, No. (%)	103/507 (20.3)	42 (21.1)	51/244 (20.9)	10 (15.6)
Anticoagulant (alone or combined), No. (%)	16/507 (3.2)	5 (2.5)	9/244 (3.7)	2 (3.1)
Baseline cerebrovascular imaging				
Any CMB, No./total (%)	156/505 (30.9)	59/197 (29.9)	73/244 (29.9)	24/64 (37.5)
CMB count, median (IQR)	0 (0‐1)	0 (0‐1)	0 (0‐1)	0 (0‐1)
CMB location, No./total (%)				
Lobar only	131/505 (25.9)	49/197 (24.9)	61/244 (25.0)	21/64 (32.8)
Mixed (lobar and deep)	16/505 (3.2)	7/197 (3.6)	6/244 (2.5)	3/64 (4.7)
Deep only	8/505 (1.6)	2/197 (1.0)	6/244 (2.5)	0/64 (0.0)
CMB > 4, No./total (%)	17/505 (3.4)	7/197 (3.6)	8/244 (3.3)	2/64 (3.1)
Fazekas D + P sum, median (IQR)	2 (2‐3)	2 (2‐3)	2 (2‐3)	2 (2‐3)
Any cSS, No./total (%)	2/503 (0.4)	2/197 (1.0)	0/242 (0)	0/64 (0)
Any lacune, No./total (%)	62/506 (12.3)	28/197 (14.2)	31/245 (12.7)	3/64 (4.7)

*Note*: The WMH burden was graded using the Fazekas scale, with deep WMH (D) and periventricular WMH (P) each scored from 0 to 3, and the summed score (D + P; range, 0‐6) was used as a continuous measure of WMH burden.

Abbreviations: AD, Alzheimer's disease; *APOE*, apolipoprotein E; CDR‐SB, Clinical Dementia Rating–Sum of Boxes; CMB, cerebral microbleed; cSS, cortical superficial siderosis; CU, cognitively unimpaired; DMT, disease‐modifying therapy; IQR, interquartile range; K‐ROAD, Korea‐Registries to Overcome Dementia and Accelerate Dementia Research; MCI, mild cognitive impairment; MMSE, Mini‐Mental State Examination; nc, non‐carrier; SD, standard deviation; WMH, white matter hyperintensity.

### 3.2 Dose titration and estimated first‐infusion Cmax

**FIGURE 1:**
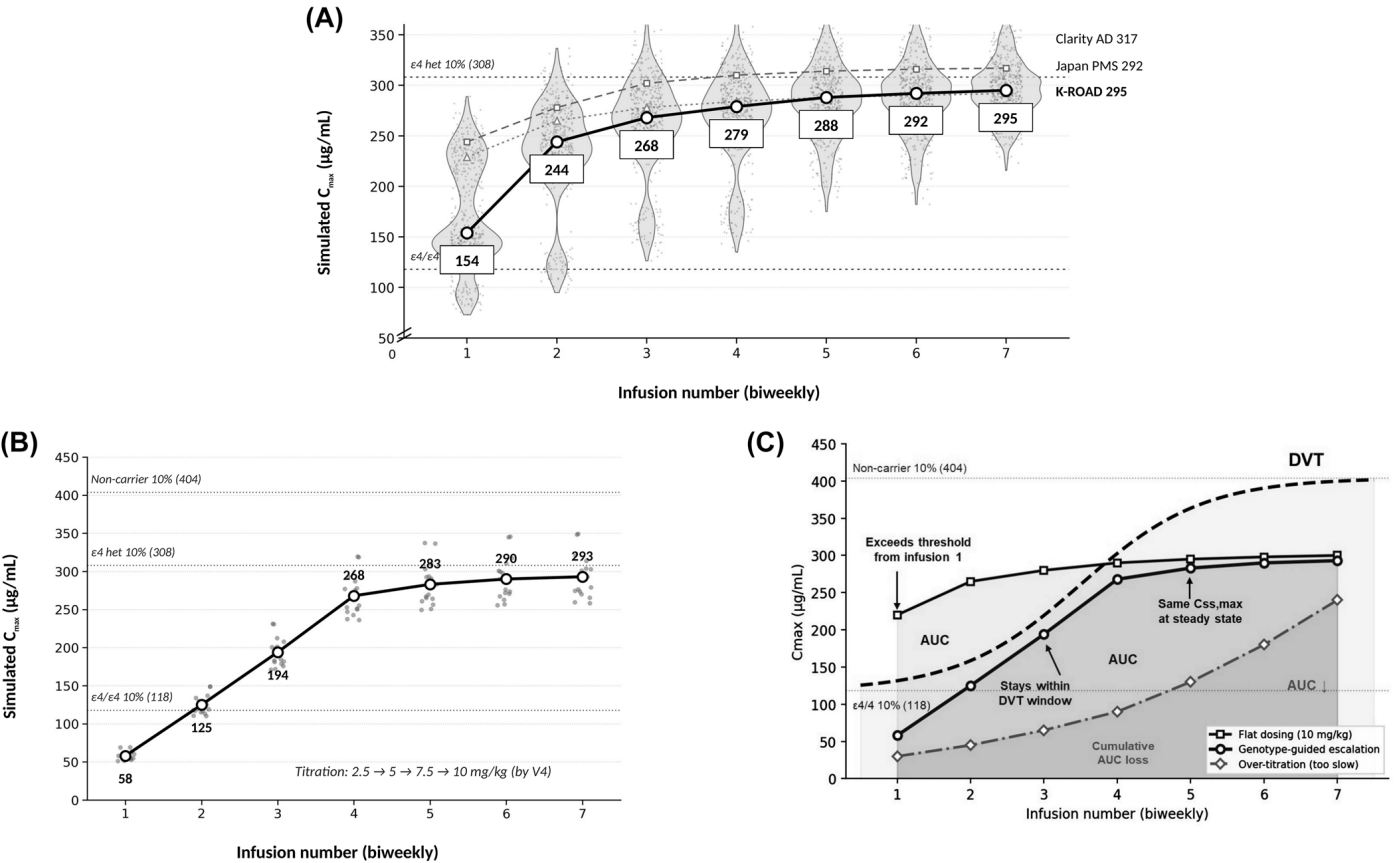
First‐infusion and steady‐state estimated Cmax trajectories under genotype‐guided dose escalation. (A) Simulated Cmax (µg/mL) across V1–V7 in K‐ROAD (*N* = 508), overlaid on Clarity AD and Japan PMS reference distributions; numerals, visit‐wise means. Dashed lines, *APOE*‐specific upper exposure thresholds (10th‐percentile ceiling under flat 10 mg/kg in Clarity AD). (B) Individual Cmax trajectories under K‐ROAD escalation (2.5 → 5 → 7.5 → 10 mg/kg by V4) in the Samsung Medical Center ε4/ε4 homozygote subgroup (*n* = 16); numerals, means; dashed lines, genotype‐specific ceilings. (C) Schematic of DVT window: Flat dosing exceeds threshold from V1; genotype‐guided escalation remains within DVT while converging to the same Css, max; over‐titration yields cumulative AUC loss. *APOE*, apolipoprotein E; AUC, area under the curve; Cmax, maximum plasma concentration; Css, max, steady‐state Cmax; DVT, dynamic vascular tolerance; K‐ROAD, Korea‐Registries to Overcome Dementia and Accelerate Dementia Research; PMS, post‐marketing surveillance; V1–V7, biweekly infusion visits 1–7.

All 64 ε4/ε4 homozygotes (100%) and 178 of 245 heterozygotes (72.7%) received reduced initial dosing beyond the standard labeled titration, whereas only a minority of non‐carriers received extended escalation. First‐infusion estimated Cmax, derived from the Majid et al. population PK model,^5^ remained below the genotype‐specific 10% ARIA‐E risk thresholds (118, 308, and 404 µg/mL for ε4/ε4 homozygotes, heterozygotes, and non‐carriers, respectively) in all 508 patients, with mean first‐infusion Cmax values of 93 µg/mL in ε4/ε4 homozygotes, 146 µg/mL in heterozygotes, and 230 µg/mL in non‐carriers. With subsequent titration, mean estimated Cmax rose progressively at infusions 2 through 7 (ε4/ε4 homozygotes: 121, 161, 172, 223, 232, and 275 µg/mL; heterozygotes: 235, 264, 274, 288, 292, and 296 µg/mL; non‐carriers: 268, 287, 292, 297, 297, and 299 µg/mL). Heterozygotes and non‐carriers remained below their respective thresholds at all seven infusions, whereas the mean Cmax in ε4/ε4 homozygotes exceeded the 118‐µg/mL threshold from infusion 2 onward, approaching the Clarity AD steady‐state value of 317 µg/mL only by infusion 7 (Figure 1A; eTable S3). The observed per‐protocol titration trajectory in the Samsung Medical Center (SMC) ε4/ε4 subgroup (*n* = 16) is shown in Figure 1B. Per‐center actual administered V1–V7 dose patterns by *APOE* genotype subgroup are presented in eTable S1.

### 3.3 Infusion‐related reactions

The IRRs occurred in 149 of 508 patients (29.3%), with 95 (18.7%) occurring after the first infusion. By CTCAE grade, 54 patients (36.2% of IRR cases) had grade 1, 87 (58.4%) had grade 2, and eight (5.4%) had grade 3 events, with grades 1 and 2 together accounting for 141 of 149 cases (94.6%). Incidence did not differ significantly by *APOE* ε4 genotype (20.3% [13/64] in ε4/ε4 homozygotes, 30.2% [74/245] in heterozygotes, and 31.2% [62/199] in non‐carriers; *p* = 0.23). Per‐infusion incidence and symptom distribution are shown in eFigures S1 and S2.

### 3.4 ARIA incidence and *APOE* genotype

ARIA‐E and any ARIA‐H occurred in 17 (3.3%) and 66 (13.0%) of 508 patients, respectively, with cumulative incidences of 2.8% and 10.0% by visit 5 and 3.3% and 13.0% by visit 7 (eFigure S3A and B). Isolated ARIA‐H (without concurrent ARIA‐E) accounted for 54 events (10.6% of the cohort). Across events, 94.1% of ARIA‐E and 89.7% of ARIA‐H were radiologically mild or moderate; 95.6% of ARIA‐H were clinically asymptomatic or mild; no ARIA‐related deaths occurred (eTable S4).

**FIGURE 2:**
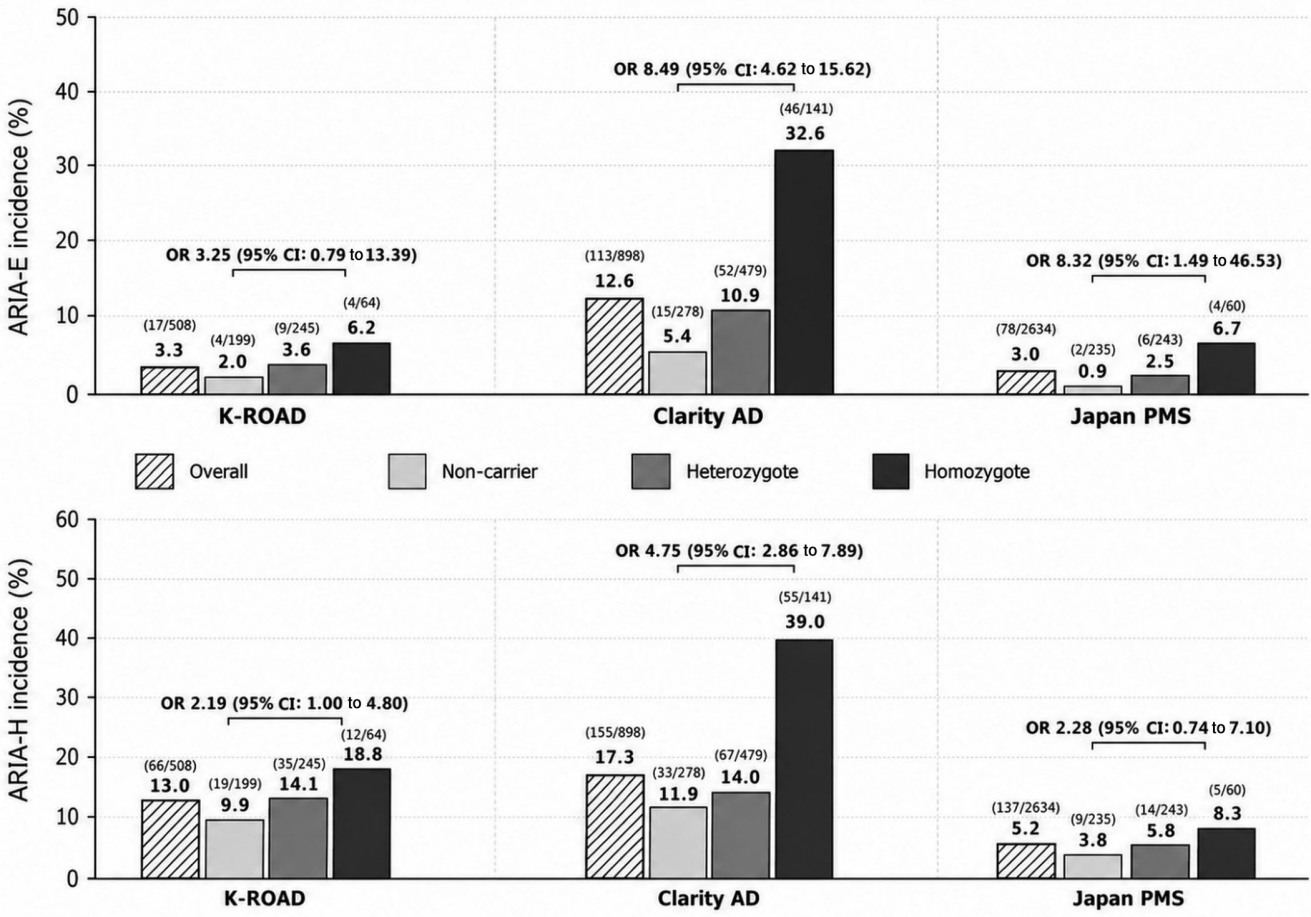
ARIA‐E and ARIA‐H incidence by *APOE* ε4 genotype across K‐ROAD, Clarity AD, and the Japan PMS cohort. (A) ARIA‐E incidence (%). (B) ARIA‐H incidence (%). Bars show overall, non‐carrier, heterozygote, and homozygote within each cohort; numerators/denominators above each bar. Brackets span Non‐carrier→Homozygote with the unadjusted OR (95% CI) for homozygote versus non‐carrier. Clarity AD from van Dyck et al.^1^; Japan PMS from Ihara et al. (2025). *APOE*, apolipoprotein E; ARIA‐E, amyloid‐related imaging abnormalities‐edema/effusion; ARIA‐H, amyloid‐related imaging abnormalities–hemosiderin; CI, confidence interval; K‐ROAD, Korea‐Registries to Overcome Dementia and Accelerate Dementia Research; OR, odds ratio; PMS, post‐marketing surveillance.

Within K‐ROAD, ARIA‐E incidence was 2.0% (4 of 199) in non‐carriers, 3.7% (9 of 245) in heterozygotes, and 6.2% (4 of 64) in ε4/ε4 homozygotes – approximately three times higher in homozygotes than in non‐carriers – while any ARIA‐H incidence was 9.5% (19 of 199), 14.3% (35 of 245), and 18.8% (12 of 64), respectively (Figure 2A). The K‐ROAD ε4/ε4‐versus‐non‐carrier OR for ARIA‐E (3.25; 95% CI: 0.79 to 13.39) was numerically lower than those reported in Clarity AD (8.49; 95% CI: 4.62 to 15.62)^1^ and Japan PMS (8.32; 95% CI: 1.49 to 46.53)^21^; given the small number of ε4/ε4 ARIA‐E events in K‐ROAD (*n* = 4) and the wide and overlapping confidence intervals across cohorts, this difference should be regarded as a numerical observation rather than a statistically significant divergence (Figure 2B). A post hoc detectable‐effect analysis indicated that, given 64 ε4/ε4 homozygotes and an observed non‐carrier ARIA‐E rate of 2.0%, the minimum detectable OR at 80% power was approximately 6.1 (Fisher exact test); the observed K‐ROAD OR of 3.25 corresponded to an achieved power of approximately 37%.

### 3.5 Pre‐existing cerebrovascular burden and ARIA‐H

Baseline cerebrovascular burden was elevated in ARIA‐H‐containing phenotypes: median CMB count was 0 (IQR: 0 to 1) in patients with no ARIA, 0 (0‐0) in isolated ARIA‐E, 2 (0 to 3) in isolated ARIA‐H (*p* < 0.001 vs no ARIA), and 1.5 (0 to 4.8) in combined ARIA‐E plus ARIA‐H (*p* = 0.002 vs no ARIA), with baseline WMH paralleling this pattern (eTable S5).^9^, ^10^, ^11^ Any ARIA‐H was therefore prespecified as the primary outcome for multivariable analysis, with isolated ARIA‐H examined as a secondary analysis.

**FIGURE 3:**
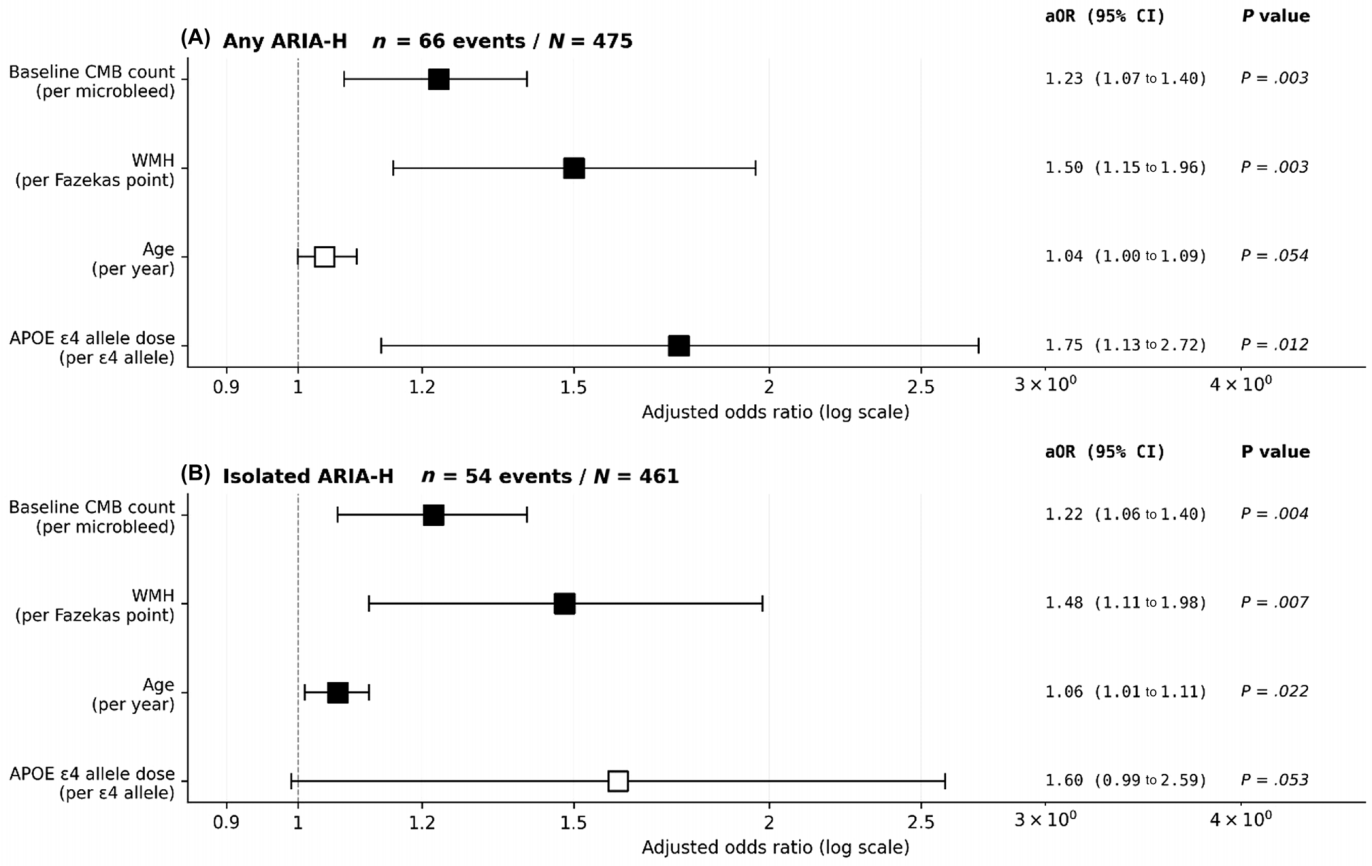
Multivariable logistic regression forest plot for any ARIA‐H (A) and isolated ARIA‐H (B) in K‐ROAD. Squares indicate aOR and horizontal bars the 95% CI (log scale); filled squares, *p* < 0.05; open squares, non‐significant. Each covariate is adjusted for all others in the same model. Isolated ARIA‐H excludes patients with concurrent ARIA‐E. Vertical dashed line, aOR = 1. aOR, adjusted odds ratio; *APOE*, apolipoprotein E; ARIA‐E, amyloid‐related imaging abnormalities‐edema/effusion; ARIA‐H, amyloid‐related imaging abnormalities‐hemosiderin; CI, confidence interval; CMB, cerebral microbleed; K‐ROAD, Korea‐Registries to Overcome Dementia and Accelerate Dementia Research; WMH, white matter hyperintensity.

Univariate analyses (eTable S6) identified baseline CMB count, Fazekas D+P sum, age, and *APOE* ε4 allele dose as significantly associated with any ARIA‐H (*p* < 0.1 each); these four covariates were carried forward into the multivariable model for any ARIA‐H and applied to isolated ARIA‐H as parallel comparisons. In multivariable logistic regression for any ARIA‐H (*n* = 66), baseline CMB count (aOR, 1.23 per microbleed; 95% CI: 1.07 to 1.40; *p* = 0.003), WMH (aOR, 1.50 per Fazekas point; 95% CI: 1.15 to 1.96; *p* = 0.003), and *APOE* ε4 allele dose (aOR, 1.75 per ε4 allele; 95% CI: 1.13 to 2.72; *p* = 0.012) were independently associated with risk, while the association for age (aOR, 1.04 per year; 95% CI: 1.00 to 1.09; *p* = 0.054) was of borderline statistical significance (Figure 3A). In the secondary analysis restricted to isolated ARIA‐H (*n* = 54), baseline CMB count (aOR, 1.22 per microbleed; 95% CI: 1.06 to 1.40; *p* = 0.004), WMH (aOR, 1.48 per Fazekas point; 95% CI: 1.11 to 1.98; *p* = 0.007), and age (aOR, 1.06 per year; 95% CI: 1.01 to 1.11; *p* = 0.022) remained independent predictors, whereas the association for *APOE* ε4 allele dose was of borderline statistical significance (aOR, 1.60 per ε4 allele; 95% CI: 0.99 to 2.59; *P* = 0.053) (Figure 3B).

Prespecified sensitivity analyses confirmed the robustness of these findings. Cluster‐robust standard errors clustered by clinical site (eight centers) yielded identical point estimates, with Fazekas D+P sum and *APOE* ε4 allele dose remaining statistically significant; site‐stratified Mantel‐Haenszel analyses showed no heterogeneity in ARIA‐E incidence across centers (*p* = 0.127; Breslow‐Day *p* = 0.85; eTable S7), indicating that center‐level clustering did not materially modify the primary ARIA‐H findings. A separate sensitivity analysis substituting lobar versus deep CMB count for total CMB count in the multivariable model showed that lobar CMB count remained an independent predictor of any ARIA‐H (aOR 1.25; 95% CI: 1.08 to 1.46; *p* = 0.003) and isolated ARIA‐H (aOR 1.26; 95% CI: 1.08 to 1.47; *p* = 0.003), whereas deep CMB count alone showed no independent association in either outcome (any ARIA‐H: aOR 0.86; 95% CI: 0.39 to 1.89; *p* = 0.70; isolated ARIA‐H: aOR 0.44; 95% CI: 0.08 to 2.25; *p* = 0.32; eTable S8). Lobar microbleeds predominated overall (84.5% of CMB‐positive patients had lobar‐only distribution; eTable S5) and across all ARIA‐H‐containing phenotypes (isolated ARIA‐H: 59.3% lobar‐only with no deep‐only cases; combined ARIA‐E + ARIA‐H: 41.7% lobar‐only), consistent with CAA as the predominant structural substrate.

## 4 DISCUSSION

In this multicenter prospective cohort study, we implemented an *APOE* ε4 genotype‐guided dose‐escalation protocol that minimized first‐infusion Cmax and, through gradual up‐titration, may create a time window during which one or more candidate mechanisms – including a putative dynamic vascular tolerance (DVT) effect, reduced cumulative cerebrovascular load of solubilized Aβ – could attenuate the acute drug‐vascular interaction underlying ARIA‐E. Under this strategy, the OR for ARIA‐E in *APOE* ε4 homozygotes relative to non‐carriers was numerically lower than in Clarity AD and Japan PMS. In contrast, ARIA‐H was independently predicted by baseline vascular burden with an isolated ARIA‐H incidence comparable to Clarity AD. These findings dissociate the two ARIA subtypes along distinct mechanistic axes: ARIA‐E as a transient, PK‐driven drug‐vascular interaction potentially mitigated by gradual up‐titration through a putative DVT mechanism, and ARIA‐H as a baseline cerebrovascular vulnerability – predominantly reflecting CAA rather than hypertensive small‐vessel disease – that is largely independent of titration schedule, raising the possibility of a dual‐axis risk‐management framework – genotype‐guided escalation for ARIA‐E and baseline MRI screening for CAA‐related cerebrovascular vulnerability for ARIA‐H.

Our observations are relevant to how lecanemab might be scheduled in real‐world practice. The *APOE* ε4 homozygote‐versus‐non‐carrier OR for ARIA‐E (OR: 3.25; 95% CI: 0.79 to 13.39) was numerically lower than those reported in Clarity AD (OR: 8.49)^1^ and Japan PMS (OR: 8.32),^21^ though the wide and overlapping confidence intervals preclude interpretation as a statistically significant cross‐cohort divergence. Although the modest homozygote event count (*n* = 4) limits precision (post hoc minimum detectable OR ≈ 6.1 at 80% power), the directionally consistent attenuation observed is compatible with an effect under this protocol; causal effectiveness, however, cannot be established from these observational data. If real, this pattern may reflect one or more candidate mechanisms operating under gradual exposure. A putative DVT effect – in which gradual up‐titration allows cerebrovascular adaptation to progressively increasing antibody‐amyloid binding at vessel walls before steady‐state exposure is reached (Figure 1C) – is one possibility. Because the absolute Cmax gap between ε4/ε4 homozygotes and non‐carriers is largest at the initial infusions, homozygotes would derive the greatest hypothesized protection from a DVT‐inducing schedule, plausibly explaining the more compressed genotype gradient observed. An alternative or complementary mechanism is a reduced cumulative cerebrovascular load of solubilized Aβ under gradual titration, also limiting the acute vasogenic edema reaction central to ARIA‐E. These mechanisms are not mutually exclusive, and their relative contribution cannot be distinguished from this observational dataset. Direct mechanistic biomarkers (e.g., blood‐brain barrier permeability imaging or dynamic contrast‐enhanced MRI) were not assessed in this real‐world cohort; mechanistic confirmation will require dedicated translational studies. Notably, none of these candidate mechanisms would be expected to materially modify ARIA‐H risk – a dissociation that itself may be informative. ARIA‐H is hypothesized to reflect acute perturbation of a chronic structural cerebrovascular substrate (CAA and small‐vessel disease established over years), which is unlikely to be modifiable by acute PK adjustment, whereas ARIA‐E reflects an acute Cmax‐dependent disruption of vascular permeability that could plausibly be mitigated by gradual exposure. These hypothesized distinct temporal scales raise the possibility of the dual‐axis safety framework outlined below.

ARIA‐H in K‐ROAD was not clearly reduced by titration in cross‐cohort comparisons and appeared instead to be governed by pre‐existing cerebrovascular vulnerability.^12^, ^13^, ^28^ Unlike ARIA‐E, the *APOE* ε4/ε4‐versus‐non‐carrier OR for any ARIA‐H was numerically similar between K‐ROAD and Japan PMS (2.19 vs 2.28)^21^ despite their different titration schedules. However, cross‐cohort comparison alone cannot establish a causal absence of titration effect on ARIA‐H, given confounding by population, imaging, and baseline characteristics; within‐study designs analogous to the TRAILBLAZER‐ALZ 6^7^ would be required for definitive testing. The higher any ARIA‐H OR in the total Clarity AD lecanemab arm (4.75) largely reflects concurrent ARIA‐E in homozygotes (32.6%); when restricted to isolated ARIA‐H, the homozygote‐versus‐non‐carrier OR converges (K‐ROAD 1.75, Clarity AD Core 1.60).^1^, ^29^ Baseline vascular burden – indexed by CMB count^9^, ^10^, ^11^ and moderate to severe WMHs,^20^, ^24^, ^25^ with age contributing borderline – constituted the dominant independent predictor set on multivariable modeling. Location‐specific sensitivity refined this signal: lobar CMB count remained the independent CMB predictor (aOR: 1.25, *p* = 0.003), whereas deep CMB count showed no independent association. Because lobar microbleeds are established CAA markers and deep microbleeds reflect hypertensive arteriopathy, these findings implicate CAA – rather than hypertensive small‐vessel disease – as the predominant structural substrate. This is reinforced by lobar predominance in K‐ROAD (84.5% lobar‐only among CMB‐positive patients), paralleling prior reports in amyloid‐positive AD/MCI cohorts (∼75% to 90% lobar predominance).^30^, ^31^, ^32^ Strikingly, *APOE* ε4 allele dose retained a substantial independent association with any ARIA‐H (aOR: 1.75 per allele)^14^, ^15^ despite the genotype‐adapted protocol, suggesting that the genotype‐linked hemorrhagic signal was unlikely to be neutralizable by PK modulation and did not operate through the acute drug‐vascular pathway attenuated by DVT. This mechanistic separation suggests that ARIA‐H risk should be considered before treatment initiation through baseline MRI‐based screening, consistent with a hypothesis‐generating dual‐axis framework in which titration intensity is matched to *APOE*‐defined ARIA‐E vulnerability while eligibility is governed by MRI‐based ARIA‐H vulnerability. The single clinically severe event in our cohort – an *APOE* non‐carrier with severe baseline periventricular WMH and concomitant antiplatelet use who developed intracerebral hemorrhage after the first infusion – underscores the clinical importance of baseline screening, even in patients without *APOE* ε4 carriage (eTable S4).

IRR represented a distinct tolerability axis mechanistically separate from ARIA. Although we initially considered whether IRR would track the genotype‐dependent Cmax gradient that shaped ARIA‐E, the IRR incidence in K‐ROAD (149 of 508; 29.3%) was comparable to that of the pivotal Clarity AD trial (∼26%)^1^ and higher than the Japan PMS cohort (∼17%; *n* = 2672)^21^ – consistent with the prospective, structured surveillance inherent in K‐ROAD – and showed no association with *APOE* ε4 allele dose, arguing against a PK mechanism and supporting an immune‐mediated etiology. IRR should therefore be managed through universal premedication and early‐infusion monitoring, independent of *APOE*‐ or vascular‐burden‐based stratification.

For international readership, two K‐ROAD design features warrant emphasis. First, the Korean Appropriate Use Recommendations treat >4 baseline microbleeds, cSS, and anticoagulant use as relative rather than absolute exclusions, capturing a population with higher baseline cerebrovascular vulnerability than US trial cohorts. Second, SWI was used for ARIA‐H surveillance, which detects substantially more microbleeds than the GRE protocols in Clarity AD and Japan PMS.^21^, ^22^ Despite the fact that both factors were expected to raise observed ARIA‐H rates, K‐ROAD's isolated ARIA‐H incidence (10.6%) remained comparable to the total Clarity AD lecanemab arm (8.9%), supporting the interpretation that ARIA‐H risk is governed primarily by baseline cerebrovascular vulnerability rather than by inclusion threshold or detection sensitivity alone.

A principal strength of this study is its purpose‐built multicenter design applying clinical trial‐level monitoring rigor to real‐world lecanemab dosing, occupying a distinct evidence niche between regulatory trials and conventional PMS. Several limitations nonetheless warrant acknowledgment. First, the observational design, between‐site heterogeneity, and modest ε4/ε4 subgroup (*n* = 64) preclude definitive causal attribution of the attenuated ARIA‐E gradient to the protocol, although the biologically coherent pattern across K‐ROAD, Clarity AD, and Japan PMS mitigates this concern. Second, IRB approval frameworks differed across centers (prospective enrollment at the coordinating center; retrospective ascertainment elsewhere under harmonized standards), potentially introducing site‐level variability. Third, follow‐up was confined to the core titration window (first seven infusions); long‐term safety under maintenance dosing remains uncharacterized. Fourth, MRI sequence heterogeneity affected comparisons: Across cohorts, K‐ROAD employed SWI, whereas Japan PMS relied on GRE protocols,^22^, ^23^ likely contributing to lower CMB detection in Japan PMS; within K‐ROAD, per‐center sample sizes precluded formal stratified analyses by MRI protocol (eTable S7). Fifth, the population PK simulation relied on a published two‐compartment model^5^, ^6^ rather than participant‐level plasma measurements, limiting the precision of Cmax estimates. Sixth, the proposed DVT mechanism remains a working hypothesis; direct biomarkers of vascular adaptation were not assessed in this real‐world cohort, and mechanistic confirmation will require dedicated studies. Seventh, in the absence of a within‐sample untitrated comparator, the inference that titration does not modify ARIA‐H risk relies on cross‐cohort comparison with Japan PMS, which is subject to confounding by population, imaging, and selection differences; a randomized titration trial analogous to TRAILBLAZER‐ALZ 6 will be required for definitive confirmation. Eighth, ARIA raters were not formally blinded to *APOE* genotype; although this could introduce expectation bias, the absence of inflation in ε4/ε4 ARIA rates relative to centrally blinded cohorts (e.g., Clarity AD) argues against systematic upward bias. Finally, our findings derive from a single East Asian cohort, requiring external validation before broader generalization. Despite these limitations, our data provide the first prospective real‐world evidence that an *APOE* ε4 genotype‐guided dose‐escalation protocol is associated with a numerically attenuated genotype‐dependent ARIA‐E gradient while leaving structurally determined ARIA‐H risk largely unchanged, raising the hypothesis of a dual‐axis safety framework for real‐world lecanemab deployment.

In conclusion, K‐ROAD observations are compatible with attenuation of the *APOE* ε4‐dependent ARIA‐E gradient under genotype‐guided dose‐escalation, whereas structurally determined ARIA‐H risk remained largely unchanged. These findings raise the hypothesis of a dual‐axis safety framework – integrating *APOE*‐guided titration with MRI‐based vascular‐burden screening – that warrants prospective evaluation before broader real‐world implementation.

## CONFLICT OF INTEREST STATEMENT

All authors declare no conflicts of interest. Author disclosures are available in the Supporting Information.

## CONSENT STATEMENT

Written informed consent was obtained from all participants or their legally authorized representatives. Ethics approval for this multicenter study was obtained from the IRB of each participating center. The study was conducted in accordance with the Declaration of Helsinki.

## Supporting information




**Supporting Information**: alz71765‐sup‐0001‐SuppMat.docx

**Supporting Information**: alz71765‐sup‐0002‐SuppMat.pdf
