# Supplementary material for: APOE‐guided lecanemab dosing dissociates ARIA‐E from ARIA‐H: The K‐ROAD study
Source: Alzheimers Dement. 2026 Aug 25;22(8):e71765. doi: 10.1002/alz.71765 (PMC13507511; doi:10.1002/alz.71765)
Supplement: Supplementary file 2 [file ALZ-22-e71765-s002.docx]

**Supplementary Materials**

**eFigure 1.** Per-infusion incidence of infusion-related reactions by CTCAE grade. Bars show the proportion of infusions (denominators annotated) with IRRs of each grade at each scheduled infusion. Abbreviations: CTCAE, Common Terminology Criteria for Adverse Events; IRR, infusion-related reaction.

**eFigure 2.** Symptom distribution in patients with infusion-related reactions (n = 149). Bars show the proportion of IRR-affected patients reporting each symptom category; a single patient could report multiple symptoms. Abbreviations: IRR, infusion-related reaction.

**eFigure 3.** Cumulative incidence of ARIA-E (A) and any ARIA-H (B) at V5 and V7. V5 and V7 denote scheduled MRI assessments obtained before the 5th and 7th biweekly lecanemab infusions, respectively. Abbreviations: ARIA-E, amyloid-related imaging abnormalities with edema or effusion; ARIA-H, ARIA with hemosiderin deposition; MRI, magnetic resonance imaging; V5 / V7, visit 5 / visit 7.

**eTable** **1**. Actual mean administered titration pattern (mg/kg) across V1→V7 biweekly infusions by APOE genotype subgroup and participating center (K-ROAD, N = 508)

| Center (N) | APOE ε4/ε4 | APOE ε4 heterozygote | Noncarrier |
| --- | --- | --- | --- |
| Samsung Medical Center (119) | 2.5→5→7.5→10→10→10→10 (n=16) | 5→10→10→10→10→10→10 (n=54) | 8.5→10→10→10→10→10→10 (n=49) |
| HappyMind Clinic (154) | 4→4→6.5→6.5→8→8→10 (n=17) | 6.5→6.5→8.5→8.5→10→10→10 (n=75) | 9.5→9.5→10→10→10→10→10 (n=62) |
| Asan Medical Center (120) | 5→5→5→5→5→5→5 (n=16) | 10→10→10→10→10→10→10 (n=58) | 10→10→10→10→10→10→10 (n=46) |
| Chonnam National Univ. (40) | 3.5→3.5→4→4.5→6.5→6.5→7.5 (n=6) | 4→4→5→5.5→7→7.5→8 (n=15) | 4→5→6→6.5→7.5→8→8 (n=18) |
| Pusan National Univ. (26) | 4→4→5→5→7.5→7.5→8.5 (n=4) | 6→6→7.5→7.5→9.5→9.5→9.5 (n=13) | 8→8→9→9→9.5→9.5→10 (n=7) |
| Haeundae Paik Hospital (17) | 4→4→6→6→7.5→7.5→9.5 (n=3) | 5.5→6→7.5→8→9→9→9.5 (n=9) | 5.5→7→8→8→9→9→9 (n=5) |
| Yongin Severance (16) | 2.5→2.5→5→5→8→8→10 (n=1) | 8.5→8.5→9.5→9→9→9→9 (n=9) | 10→10→10→10→10→10→10 (n=6) |
| Kyung Hee Univ. Med Ctr. (16) | N/A (n=0) | 9→9→9→9→9→9→9 (n=10) | 9→9→9→9→9→9→9 (n=6) |

Abbreviations: APOE, apolipoprotein E; CMB, cerebral microbleed; mg/kg, milligram per kilogram body weight; V1–V7, biweekly infusions 1 through 7.

Notes. The table presents actual mean administered dose (rounded to the nearest 0.5 mg/kg) at each biweekly infusion (V1–V7) by APOE genotype subgroup; APOE ε4 heterozygotes are reported as a combined group regardless of baseline CMB status. The reference protocol proposed by the coordinating center (Samsung Medical Center) specified initial dose escalation starting at 2.5 mg/kg for APOE ε4/ε4 homozygotes, 5 mg/kg for ε4 heterozygotes with baseline CMBs, and 10 mg/kg for ε4 heterozygotes without CMBs and noncarriers, escalating to a maintenance dose of 10 mg/kg biweekly. All 64 APOE ε4/ε4 homozygotes (100%) received reduced initial dosing (≤5 mg/kg at V1) regardless of center, consistent with this principle, and no center exceeded the protocol-specified maximum of 10 mg/kg in any subgroup. Real-world adaptation primarily involved the pace of escalation in heterozygotes and noncarriers. These center-level variations were addressed in sensitivity analyses (eTable 7, eTable 8) and did not materially modify the primary findings of APOE-guided ARIA-E mitigation or the dissociation of ARIA-H risk from drug exposure.

**eTable 2.** MRI acquisition protocols by center

| Center | Scanner (manufacturer, field strength) | Sequences (FLAIR / SWI or T2* / DWI / T1) | Slice thickness, mm | Monitoring schedule |
| --- | --- | --- | --- | --- |
| Samsung Medical Center | Philips / GE, 3.0T | FLAIR, SWI, DWI, 3D T1 | 3.0–5.0 | Baseline, V5, V7 |
| HappyMind Clinic | Site-specific, 1.5T or 3.0T | FLAIR, SWI or T2*, DWI, T1 | 2.0 | Baseline, V5, V7 |
| Asan Medical Center | Philips, 3.0T | FLAIR, SWI, DWI, 3D T1 | 1.12–2 | Baseline, V5, V7 |
| Chonnam National University Hospital | Siemens / Philips, 3.0T | FLAIR, SWI, DWI, T1 | 4.0 | Baseline, V5, V7 |
| Pusan National University Hospital | Siemens 3.0T | FLAIR, SWI, DWI | 2.0–5.0 | Baseline, V5, V7 |
| Yongin Severance Hospital | Site-specific, 3.0T | FLAIR, SWI, DWI, T1 | 1.0 | Baseline, V5, V7 |
| Haeundae Paik Hospital | Site-specific, 3.0T | FLAIR, SWI, DWI, T1 | 1.0–2.0 | Baseline, V5, V7 |
| Kyung Hee University Hospital | Site-specific, 3.0T | FLAIR, SWI, DWI, T1 | 2.0–5.0 | Baseline, V5, V7 |

Abbreviations: ADRD-TWG, Alzheimer Disease and Related Disorders Therapeutics Working Group; ARIA, amyloid-related imaging abnormalities; DWI, diffusion-weighted imaging; FLAIR, fluid-attenuated inversion recovery; MRI, magnetic resonance imaging; SWI, susceptibility-weighted imaging; T1, T1-weighted imaging; T2*, T2-star weighted imaging; V5, visit 5; V7, visit 7.

Notes. V5 and V7 denote scheduled MRI assessments obtained before the 5th and 7th biweekly lecanemab infusions, respectively. All 8 centers uniformly performed additional post-ARIA follow-up MRIs to confirm ARIA-E resolution and ARIA-H stability before resuming lecanemab dosing, per the ADRD-TWG Appropriate Use Recommendations and Korean Dementia Association guidelines.

**eTable 3.** Simulated lecanemab C_max_ across the first 7 biweekly infusions, by cohort and APOE ε4 genotype

| Cohort | Mean BWᵃ  (kg) | Simulated C_max_ (μg/mL) by infusion number (biweekly) | | | | | | |
| --- | --- | --- | --- | --- | --- | --- | --- | --- |
|  |  | 1 | 2 | 3 | 4 | 5 | 6 | 7 |
| Clarity ADᵇ (lecanemab arm) (n = 898) | 72.0 | 244 | 278 | 302 | 310 | 314 | 316 | 317 |
| Japan PMSᶜ (n = 2,672) | 53.5 | 229 | 265 | 278 | 284 | 288 | 290 | 292 |
| K-ROAD overall (n = 508) | 58.4 | 154 | 244 | 268 | 279 | 288 | 292 | 295 |
| APOE ε4/ε4 (homozygote) (n = 64) | 57.2 | 93 | 121 | 161 | 172 | 223 | 232 | 275 |
| APOE ε4 heterozygote (n = 249) | 57.8 | 146 | 235 | 264 | 274 | 288 | 292 | 296 |
| APOE ε4 noncarrier (n = 202) | 59.6 | 230 | 268 | 287 | 292 | 297 | 297 | 299 |
| SMC APOE ε4/ε4 (per-protocol titration)ᵈ (n = 16) | 58.6 | 58 | 125 | 194 | 268 | 283 | 290 | 293 |
|  |  |  |  |  |  |  |  |  |

Abbreviations: APOE, apolipoprotein E; BW, body weight; Cmax, maximum plasma concentration; K-ROAD, Korean Real-world Observation of Anti-amyloid Drug; PMS, post-marketing surveillance; popPK, population pharmacokinetic; SMC, Samsung Medical Center; V₁, central compartment volume of distribution; V1–V7, biweekly infusion visits 1–7.

Notes. Cmax values were simulated using the published 2-compartment population pharmacokinetic model of lecanemab (Majid et al., 2024), with cohort-specific body weight, dose, and infusion timing as inputs.

ᵃ Mean body weight by cohort.

ᵇ Clarity AD Open-label Extension cohort.

ᶜ Japan post-marketing surveillance cohort.

ᵈ SMC ε4/ε4 subgroup of K-ROAD under cautious escalation (2.5 → 5 → 7.5 → 10 mg/kg by V4); underlies Figure 1B.

**eTable 4.** Radiologic and clinical severity of ARIA-E and ARIA-H events

| Clinical grade (CTCAE) | ARIA-E (n = 17) | | | ARIA-H events (n = 69)^b^ | | |
| --- | --- | --- | --- | --- | --- | --- |
|  | Radiologic severityᵃ | | | Radiologic severityᵃ | | |
|  | Mild | Moderate | Severe | Mild | Moderate | Severe |
| Asymptomatic | 10 | 6 | 0 | 57 | 3 | 2 |
| Mild | 0 | 0 | 0 | 1 | 1 | 2 |
| Moderate | 0 | 0 | 1 | 0 | 0 | 2 |
| Severe | 0 | 0 | 0 | 0 | 0 | 1ᶜ |
| Total | 10 | 6 | 1 | 58 | 4 | 7 |

Abbreviations: ADRD-TWG, Alzheimer Disease and Related Disorders Therapeutics Working Group; ARIA-E, amyloid-related imaging abnormalities–edema/effusion; ARIA-H, amyloid-related imaging abnormalities–hemosiderin (microhemorrhage, cortical superficial siderosis, or macrohemorrhage); CTCAE, Common Terminology Criteria for Adverse Events; cSS, cortical superficial siderosis.

Notes. Sixteen of 17 ARIA-E events (94%) and 61 of 68 ARIA-H events (90%) were radiologically mild to moderate. Severe clinical events at the cohort level: ARIA-E, 1 of 508 (0.2%); ARIA-H, 7 of 508 (1.4%). No fatal ARIA events occurred.

ᵃ Radiologic severity graded per ADRD-TWG Appropriate Use Recommendations; clinical severity per CTCAE v5.0.

ᵇ ARIA-H event count (n = 68) exceeds the any-ARIA-H patient count (n = 66) because some patients had multiple subtypes (microbleed, cSS, and/or macrohemorrhage) graded separately; combined microbleed + cSS events were graded by the more severe component.

ᶜ The single clinically severe ARIA-H event occurred in an 84-year-old female APOE ε3/ε3 noncarrier with mild AD dementia, BMI 19.1, severe baseline periventricular WMH (Fazekas P = 3), no baseline microbleeds or cortical superficial siderosis, on concomitant antiplatelet therapy. Three days after the first 10 mg/kg infusion she developed headache, vomiting, and depressed consciousness; brain CT confirmed intracerebral hemorrhage. She was managed conservatively without neurosurgical intervention, and lecanemab was permanently discontinued. At last available follow-up, consciousness had not recovered and the patient had been transferred to a long-term care facility (modified Rankin Scale = 5).

**eTable 5.** Baseline cerebrovascular burden stratified by ARIA phenotype (K-ROAD, N = 508)

| Variable | No ARIA  (n = 437) | Isolated ARIA-E (n = 5) | Isolated ARIA-H  (n = 54) | Combined ARIA-E + ARIA-H (n = 12) |
| --- | --- | --- | --- | --- |
| Cerebral microbleed count (total), median (IQR)ᵃ | 0 (0–1) | 0 (0–0) | 2 (0–3)* | 1.5 (0–4.75)* |
| Lobar CMB count, median (IQR)ᵃ | 0 (0–0) | 0 (0–0) | 1 (0–3)* | 0 (0–4.75)* |
| Deep CMB count, median (IQR)ᵃ | 0 (0–0) | 0 (0–0) | 0 (0–0) | 0 (0–0) |
| CMB location, No./total (%) |  |  |  |  |
| Lobar only | 93/434 (21.4) | 1/5 (20.0) | 32/54 (59.3)* | 5/12 (41.7)* |
| Mixed (lobar and deep) | 10/434 (2.3) | 0/5 (0) | 5/54 (9.3)* | 1/12 (8.3) |
| Deep only | 7/434 (1.6) | 0/5 (0) | 0/54 (0) | 1/12 (8.3) |
| Fazekas D+P sum, median (IQR)ᵃ | 2 (2–3) | 2 (2–2) | 3 (2–4)* | 2 (2–4) |
| Lacune count, median (IQR)ᵃ | 0 (0–0) | 0 (0–0) | 0 (0–0) | 0 (0–0) |
| Any cortical superficial siderosis, No. (%)ᵇ | 2/432 (0.5) | 0/5 (0) | 0/55 (0) | 0/12 (0) |

Abbreviations: ARIA, amyloid-related imaging abnormalities; ARIA-E, ARIA with edema or effusions; ARIA-H, ARIA with cerebral microhemorrhages, cortical superficial siderosis, or macrohemorrhage; CMB, cerebral microbleed; IQR, interquartile range; WMH, white matter hyperintensity.

Notes. WMH burden was assessed as the Fazekas sum of deep and periventricular subscales (D + P; range, 0–6) and used as a continuous measure. Cerebral microbleeds were classified by anatomical location as deep (basal ganglia, thalamus, brainstem) or lobar (cortical-subcortical, including external capsule) per MARS criteria. Lobar CMB count was computed as total CMB minus deep CMB; in a small number of cases (n = 7) with apparent total/deep count discordance, deep CMB was capped at total CMB (lobar = 0). Three patients had missing baseline CMB data and are not included in the location-specific rows. Among CMB-positive patients (N = 155), 84.5% had lobar-only distribution, 10.3% had mixed, and 5.2% had deep-only distribution, with lobar predominance most pronounced in ARIA-H–containing phenotypes (isolated ARIA-H 59.3% lobar-only with no deep-only cases), consistent with cerebral amyloid angiopathy as the predominant structural substrate.

P < .05 in pairwise comparison versus the no-ARIA reference group.

ᵃ Mann-Whitney U test versus the no-ARIA group.

ᵇ Fisher exact test versus the no-ARIA group.

**eTable 6**. Univariate **associations of candidate predictors with** ARIA outcomes (K-ROAD, N = 508)

| Variable | ARIA-E (n=17)  OR (95% CI), P | Any ARIA-H (n=66)  OR (95% CI), P | Isolated ARIA-H (n=54)  OR (95% CI), P |
| --- | --- | --- | --- |
| **Demographics** |  |  |  |
| Age (per year) | 1.01 (0.95–1.07), P = .79 | **1.06 (1.03–1.10), P < .001** | **1.08 (1.04–1.13), P < .001** |
| Female sex | 2.49 (0.71–8.80), P = .16 | 1.34 (0.76–2.36), P = .32 | 1.08 (0.59–1.96), P = .81 |
| **APOE genotype** |  |  |  |
| APOE ε4 allele dose (per allele) | **1.80 (0.89–3.64), P = .10** | **1.49 (1.02–2.18), P = .04** | 1.37 (0.90–2.07), P = .14 |
| **Vascular risk factors** |  |  |  |
| Hypertension | 0.50 (0.16–1.54), P = .23 | 0.86 (0.51–1.45), P = .56 | 1.01 (0.58–1.77), P = .98 |
| Diabetes mellitus | 0.25 (0.03–1.91), P = .18 | 0.89 (0.46–1.70), P = .72 | 1.00 (0.50–1.98), P = .99 |
| Dyslipidemia | 0.63 (0.24–1.64), P = .35 | 0.96 (0.58–1.60), P = .88 | 1.11 (0.63–1.94), P = .73 |
| **Medications** |  |  |  |
| Antiplatelet use | 0.51 (0.12–2.28), P = .38 | 1.30 (0.71–2.40), P = .40 | 1.40 (0.73–2.70), P = .31 |
| Anticoagulant use | 1.98 (0.25–15.92), P = .52 | 0.95 (0.21–4.29), P = .95 | 0.57 (0.07–4.41), P = .59 |
| **Baseline cerebrovascular imaging** |  |  |  |
| Baseline CMB count (per microbleed) | **1.22 (1.08–1.37), P = .001** | **1.39 (1.21–1.59), P < .001** | **1.35 (1.16–1.57), P < .001** |
| Fazekas D+P sum (per point) | 1.38 (0.88–2.15), P = .16 | **1.68 (1.32–2.14), P < .001** | **1.67 (1.29–2.17), P < .001** |
| Lacune count (per lacune) | 1.05 (0.52–2.14), P = .89 | 1.16 (0.81–1.66), P = .43 | 1.21 (0.83–1.76), P = .32 |

Abbreviations: APOE, apolipoprotein E; ARIA, amyloid-related imaging abnormalities; CI, confidence interval; CMB, cerebral microbleed; cSS, cortical superficial siderosis; D+P, deep + periventricular; OR, odds ratio.

Notes. Univariate logistic regression for each candidate covariate, separately for ARIA-E, any ARIA-H, and isolated ARIA-H. Variables with P < 0.1 in univariate analysis with any ARIA-H were carried forward into the multivariable model (Methods 2.8). Cortical superficial siderosis was present at baseline in only 2 patients and could not be evaluated as a univariate predictor.

**eTable 7.** Multivariable logistic regression for ARIA-H with cluster-robust standard errors clustered by clinical site (K-ROAD, N = 508)

| Covariate | Naive standard errors *aOR (95% CI), P* | Cluster-robust standard errors *aOR (95% CI), P* |
| --- | --- | --- |
| Any ARIA-H (N = 475, events = 58) | | |
| Baseline CMB count (per CMB) | 1.23 (1.07–1.40), .003 | 1.23 (0.87–1.73), .247 |
| Fazekas D+P sum (per point) | 1.50 (1.15–1.96), .003 | 1.50 (1.16–1.94), .002 |
| Age (per year) | 1.04 (1.00–1.09), .054 | 1.04 (1.01–1.07), .003 |
| APOE ε4 allele dose (per allele) | 1.75 (1.13–2.72), .012 | 1.75 (1.10–2.78), .017 |
| Isolated ARIA-H (N = 461, events = 48) | | |
| Baseline CMB count (per CMB) | 1.22 (1.06–1.40), .004 | 1.22 (0.88–1.69), .229 |
| Fazekas D+P sum (per point) | 1.48 (1.11–1.98), .007 | 1.48 (1.06–2.08), .021 |
| Age (per year) | 1.06 (1.01–1.11), .022 | 1.06 (0.99–1.13), .113 |
| APOE ε4 allele dose (per allele) | 1.60 (0.99–2.59), .053 | 1.60 (0.91–2.83), .103 |

Abbreviations: aOR, adjusted odds ratio; ARIA-E, amyloid-related imaging abnormalities with edema/effusion; ARIA-H, ARIA with microhemorrhage or superficial siderosis; CI, confidence interval; CMB, cerebral microbleed; SE, standard error.

Notes. Multivariable models were re-fit using cluster-robust (sandwich) standard errors with clinical site (8 centers) as the clustering variable to account for potential center-level confounding arising from heterogeneous titration practices. Point estimates were identical between naive and cluster-robust models by construction; only standard errors and confidence intervals differed. Fazekas D+P sum and APOE ε4 allele dose remained statistically significant under both SE specifications. The wider confidence interval for baseline CMB count under cluster-robust SE reflects between-site variability in CMB distribution. Site-stratified Mantel-Haenszel analyses showed no significant heterogeneity in ARIA-E incidence across centers (P = .127), with homogeneous APOE-ARIA-E effect across centers (Breslow-Day P = .85), supporting the inference that center-level variation in titration implementation did not materially modify ARIA-E risk. Per-center actual administered dose patterns are presented in revised eTable 1.

**eTable 8.** Sensitivity multivariable logistic regression substituting lobar versus deep cerebral microbleed count for total CMB count (K-ROAD, N = 505)

| Covariate | Lobar CMB model aOR (95% CI), P | Deep CMB model aOR (95% CI), P |
| --- | --- | --- |
| *Any ARIA-H (N = 475, events = 58)* | | |
| CMB count (location-specific, per CMB) | 1.25 (1.08–1.46), .003 | 0.86 (0.39–1.89), .70 |
| Fazekas D+P sum (per point) | 1.50 (1.15–1.96), .003 | 1.57 (1.21–2.04), .001 |
| Age (per year) | 1.04 (1.00–1.09), .053 | 1.05 (1.00–1.09), .034 |
| APOE ε4 allele dose (per allele) | 1.76 (1.14–2.74), .011 | 1.86 (1.21–2.87), .005 |
| *Isolated ARIA-H (N = 461, events = 48)* | | |
| CMB count (location-specific, per CMB) | 1.26 (1.08–1.47), .003 | 0.44 (0.08–2.25), .32 |
| Fazekas D+P sum (per point) | 1.48 (1.11–1.98), .007 | 1.53 (1.15–2.03), .003 |
| Age (per year) | 1.06 (1.01–1.11), .022 | 1.06 (1.01–1.11), .014 |
| APOE ε4 allele dose (per allele) | 1.61 (0.99–2.59), .053 | 1.76 (1.10–2.82), .018 |

Abbreviations: aOR, adjusted odds ratio; APOE, apolipoprotein E; ARIA-H, amyloid-related imaging abnormalities with microhemorrhage or superficial siderosis; CI, confidence interval; CMB, cerebral microbleed; WMH, white matter hyperintensity.

Notes. Multivariable logistic regression models substituted lobar CMB count (Lobar CMB model) or deep CMB count (Deep CMB model) for total CMB count in the primary multivariable model (Methods 2.8), adjusting for Fazekas D+P sum, age, and APOE ε4 allele dose. Lobar CMB count remained an independent predictor of both any ARIA-H and isolated ARIA-H, whereas deep CMB count alone showed no independent association in either outcome. This pattern is consistent with cerebral amyloid angiopathy as the predominant structural substrate underlying ARIA-H risk in this cohort.
